# Gender differences in the utilization of health-care services among the older adult population of Spain

**DOI:** 10.1186/1471-2458-6-155

**Published:** 2006-06-16

**Authors:** Áurea Redondo-Sendino, Pilar Guallar-Castillón, José Ramón Banegas, Fernando Rodríguez-Artalejo

**Affiliations:** 1 Department of Preventive Medicine and Public Health. School of Medicine. Universidad Autónoma de Madrid. Madrid, Spain

## Abstract

**Background:**

Compared to men, women report greater morbidity and make greater use of health-care services. This study examines potential determinants of gender differences in the utilization of health-care services among the elderly.

**Methods:**

Cross-sectional study covering 3030 subjects, representative of the non-institutionalized Spanish population aged 60 years and over. Potential determinants of gender differences in the utilization of health services were classified into predisposing factors (age and head-of-family status), need factors (lifestyles, chronic diseases, functional status, cognitive deficit and health-related quality of life (HRQL)) and enabling factors (educational level, marital status, head-of-family employment status and social network). Relative differences in the use of each service between women and men were summarized using odds ratios (OR), obtained from logistic regression. The contribution of the variables of interest to the gender differences in the use of such services was evaluated by comparing the OR before and after adjustment for such variables.

**Results:**

As compared to men, a higher percentage of women visited a medical practitioner (OR: 1.24; 95% confidence limits (CL): 1.07–1.44), received home medical visits (OR: 1.67; 95% CL: 1.34–2.10) and took ≥3 medications (OR: 1.54; 95% CL: 1.34–1.79), but there were no gender differences in hospital admission or influenza vaccination. Adjustment for need or enabling factors led to a reduction in the OR of women compared to men for utilization of a number of services studied. On adjusting for the number of chronic diseases, the OR (95% CL) of women versus men for ingestion of ≥3 medications was 1.24 (1.06–1.45). After adjustment for HRQL, the OR was 1.03 (0.89–1.21) for visits to medical practitioners, 1.24 (0.98–1.58) for home medical visits, 0.71 (0.58–0.87) for hospitalization, and 1.14 (0.97–1.33) for intake of ≥3 medications. After adjustment for the number of chronic diseases and HRQL, the OR of hospitalization among women versus men was 0.68 (0.56–0.84).

**Conclusion:**

The factors that best explain the greater utilization of health-care services by elderly women versus men are the number of chronic diseases and HRQL. For equal need, certain inequality was observed in hospital admission, in that it proved less frequent among women.

## Background

Compared to men, women live longer but, paradoxically, report greater morbidity and disability and make greater use of health-care services at the end of life [1-3]. Nevertheless, the greater utilization of health services by women is not a constant finding but depends in part on the type of service. Women tend to use preventive and diagnostic services more frequently, whereas men make greater use of emergency services [4]. Furthermore, although women are more likely than men to contact a general practitioner [5-12], when it comes to hospital admissions there is no difference [9] or, alternatively, men are hospitalized more frequently than women [13-15].

Utilization of health services by women and men differs according to the health problem for which care is required. Faced with the discovery of a lump in the armpit 2 weeks after a cold, women seek medical attention more frequently than do men, yet there are no differences in the proportion of women and men that immediately seek medical advice when a chest pain appears [16]. After adjustment for social and economic factors, women visit the medical practitioner more often than men when presenting a mood-anxiety disorder or a substance use-antisocial behaviors disorder, though the magnitude of the association between female gender and medical visit is greater in the case of the mood-anxiety disorder [17].

Various types of explanations have been postulated for the greater utilization of health-care services by women. Among these, it should be noted the women's greater need approximated by their worse state of health (greater morbidity, worse perception of health, worse health-related quality of life, and greater degree of disability than men), the different social construction of the disease (roles, attitudes, beliefs and behaviors of men and women when they are sick or worried about ill-health), which leads to different processes for seeking health care and differences between women and men in the mere provision of services [2-4].

The factors that determine gender differences in the utilization of health-care services may vary at different stages of life [4,6]. In the reproductive age, the need for gynecological care produces greater health-service use by adult women, but gender differences in utilization tend to diminish at advanced ages. Moreover, in countries with universal, cost-free health-care service coverage, greater utilization by most elderly women could largely be due to their worse state of health, particularly where curative (e.g., visits to the medical practitioner, hospitalization and use of emergency services) rather than preventive (e.g., mammography) or discretional (e.g., dental care) services are involved [13,18].

The study of the factors underlying gender differences in the utilization of health-care services among the elderly is particularly relevant. First, because this population group, whose size is progressively growing, uses these services most frequently; second, because the predominance of women over men increases with age, and health services use tend to be greater among the former. However, there are few studies with this specific objective that target representative samples of the older adult population [15,19,20]. Most research, with the exception of the study by Irizarry in Puerto Rico, comes from Anglo-Saxon countries. It is therefore possible that the results may not be applicable to countries with different socio-cultural characteristics or organization of their health services. In addition, only a few studies have specifically analyzed the influence of health-related quality of life (HRQL) on different patterns of health-services utilization by women and men [9]. Moreover, although lifestyles have been related to health service use [21], we have not found any studies linking lifestyles, such as smoking, alcohol consumption, body mass index or physical activity, with gender differences in health-care services utilization.

Accordingly, this study examines gender differences in the utilization of the principal types of health-care services among the older adult population of Spain. In addition, it identifies some of the variables contributing to such differences, including predisposing, enabling and need factors, as considered in the Andersen model of health services use [22].

## Methods

### Study design and subjects

This consisted of a cross-sectional survey covering a sample of 4008 subjects representative of the non-institutionalized Spanish population aged 60 years and over. Before conducting the interview, informed consent was obtained in all cases from subjects or cohabiting next-of-kin. The study was approved by the Clinical Research Ethics Committee of the "La Paz" University Hospital in Madrid, Spain.

Study subjects were selected through probabilistic multistage cluster sampling, stratified by region of residence and size of town. Census sections were selected at random in each cluster, followed by individual households where information was obtained from residents. Data were collected on a total of 450 census sections in Spain, with subjects being selected in two sex and three age (60–69 y, 70–79 y, 80 y and over) strata. Individuals aged 80 years and over were over-sampled to assure enough number of subjects for a meaningful analysis. Subjects were replaced for interviews only after 10 failed visits by the interviewer or because of subject's incapacity, death, institutionalization or refusal to participate. There was an overall study response rate of 71%. Reasons for non-response were 'impossible to locate after several attempts' (17%), 'refused to be interviewed' (6%) and the rest of motives (6%). Given the study's sample design, subjects were assigned a weighting coefficient according to their sex, age, region and size of town of residence, which allowed for reconstructing the characteristics of the Spanish population in the analysis.

### Study variables

Data were collected from October 2000 to February 2001 by home-based personal interview using a structured questionnaire, followed by a physical examination to measure blood pressure and anthropometric variables. Field work was undertaken by interviewers who underwent standardized training. Of the 4008 subjects interviewed, 3030 (75.58%) furnished complete information on all variables of interest for this study. The variable accounting for the largest amount of incomplete data was cognitive deficit, which could not be calculated for 414 subjects. Compared to persons who furnished complete information on the study variables, subjects who did not were more frequently women (64% versus 53%), and older (mean of 73.2 versus 71.3 years).

To obtain information on health services use individuals were asked whether they had sought medical advice in the preceding month or had received a home medical visit in the last year. They were also asked about hospital admission in the previous year, influenza vaccination in the most recent immunization campaign, and the number of medications currently being taken. Lastly, a variable of overall utilization was created which encompassed the above five variables. Individuals were deemed to have made overall use of health-care services if they used at least one of the above services.

The variables studied as possible contributing factors to gender differences in the utilization of health-care services were classified into three groups, in accordance with Andersen's classic model, i.e., predisposing, enabling and need factors [22]. Predisposing factors are defined, not as direct causes of utilization, but rather as determinants of the propensity to use such services; need can be objectively established by the medical practitioner and/or perceived by the patient; and enabling factors are conditions that enhance availability and access to services [5]. The following were studied: among the predisposing factors, age and head-of-family status; among the need factors, lifestyles variables (e.g., tobacco and alcohol consumption, sedentariness, and hypertension) were studied as indicators of objective health-needs amenable through clinical preventive services, either professional advice and counseling or drug treatment. These services could serve both for primary or secondary prevention of chronic disease (e.g. ischemic heart disease). Also, as need factors we included chronic diseases, functional status and cognitive deficit as indicators of curative and rehabilitation services, and health-related quality of life (HRQL) as an indicator of the perceived need which may contribute to seeking a specific health service; and lastly, among the enabling factors, educational level, marital status, head-of-family employment status, and social network.

Lifestyle variables on which information was obtained were tobacco use, physical activity and alcohol consumption. Moderate drinkers were defined as men who consumed ≤30 g and women who consumed ≤20 g of alcohol daily. Heavy drinkers were those who exceeded the limits of moderate alcohol consumption in each gender. In addition, weight and height were measured using standardized procedures [23]. Body mass index (BMI) was calculated as weight in kilograms divided by the square of the height in meters (kg/m^2^), and subjects were classified in three groups: low and normal weight (<25 kg/m^2^), overweight (25–29.9 kg/m^2^) and obese (≥30 kg/m^2^). Blood pressure was determined in a standardized manner [24], and subjects were deemed to be hypertensive where systolic blood pressure was ≥140 mm Hg, diastolic blood pressure was ≥90 mm Hg, or on current antihypertensive drug treatment.

Moreover, information was obtained on self-reported chronic diseases diagnosed by the physician, and specifically: asthma and chronic bronchitis, ischemic heart disease, stroke, arthritis, cataracts without treatment, diabetes mellitus, Parkinson's disease, cancer (at any site), and depression with need for treatment. These diseases are rather prevalent in people aged 60 years and older, and are important enough to be sure that people can be aware of their diagnosis by a physician. Subjects were classified into four groups: absence of disease, presence of one, two, and three or more chronic diseases.

Functional status was assessed through limitations in instrumental activities of daily living (IADL), as determined by Lawton and Brody's test [25]. This assesses limitations in the following 8 activities: using the telephone, going shopping, preparing meals, doing household chores, washing clothes, traveling independently, responsibility for own medication, and handling money. The absence of limitation in any given activity scores 1 point. Subjects deemed to be IADL-independent are women scoring 8 and men scoring 5 points, since the scores for "preparing meals", "doing household chores" and "washing clothes" are excluded in the case of men. A lower score means that the subject manifests some type of dependence, with 0 points indicating the maximum degree of dependence.

Cognitive deterioration was evaluated with the Cognition Mini-exam [26,27], which is a Spanish adaptation and validation of the original Mini-Mental Status Examination [28]. It is made up of 11 items that evaluate the following cognitive areas: orientation, memory registration, short-term recall, attention, calculation, and language comprehension and expression. The maximum score attainable is 30 and individuals who obtain a score = 22 are deemed to suffer from a cognitive deficit.

HRQL provides subjective information on the health-status of the individuals, which has been shown to correlate with morbidity. As a result, HRQL served as an indicator of perceived need of health-care services [29]. HRQL was measured using the Spanish version of the SF-36 questionnaire [30,31]. This questionnaire is made up of 36 items, which assess the following 8 HRQL components or scales: physical functioning, role-physical, bodily pain, general health, vitality, social functioning, role-emotional, and mental health. Subjects' answers to any given item receive a numerical score which, after coding, is ranked on a scale of 0 to 100, so that the higher the score the better the HRQL. After aggregating the scores for each scale, by assigning a coefficient to each of them, summary physical and mental HRQL indices were calculated.

Finally, to assess social network, subjects were asked with whom they usually lived, how often they saw family members other than those with whom they cohabited, and how often they saw friends or neighbors.

### Data analysis

Relative differences between women and men in the utilization of health-care services were summarized using odds ratios (OR) and their 95% confidence limits (CL) obtained from unconditional logistic regression, in which the principal independent variable was gender and the dependent variables were each of the health-care services studied. First, crude OR were calculated; then models were adjusted for groups of variables (predisposing, need and enabling factors). All the independent variables were modeled using dummies. The contribution of each group of variables to gender differences in the use of each health service was evaluated by comparing the OR of health services use of women versus men before and after adjustment for such groups of variables.

Analyses were performed using the SAS package, version 8.02 (2001) [32].

## Results

The study sample comprised 1672 (55.18%) women and 1358 (44.82%) men, having a mean age of 71.9 and 70.7 years, respectively.

Table 1 shows the distribution of the variables of interest by gender. Differences (p < 0.05) between women and men were in evidence for most variables, except for arterial hypertension and seeing family members. With regard to need factors for use of health-care services, women led a more sedentary lifestyle and were more frequently obese than were men, yet there were more overweight men than women. Men tended to smoke and consume excessive amounts of alcohol more frequently than did women. Compared to men, women presented with a greater number of chronic diseases, were less frequently independent in IADL, had a higher frequency of cognitive deficit, and worse HRQL in terms both of physical and mental components. With respect to enabling factors, men had a higher educational level, and a higher percentage was in paid employment than were women. Furthermore, while a greater percentage of women lived alone and saw family members daily, men nevertheless saw friends or neighbors more often than did women.

The percentage of women using health-care services was significantly higher than that of men in terms of visits to medical practitioners, home medical visits, number of medications and overall utilization (Table 2). No significant gender differences (p > 0.05) were observed in the percentage of subjects that were admitted to hospitals or received influenza vaccination.

After adjustment for predisposing factors, OR of women versus men for visits to medical practitioners, home medical visits and overall utilization of health-care services attenuated substantially and lost statistical significance (Table 3). Adjusting solely for age or head-of-family status did not yield a material reduction in the crude OR for utilization of any health-care service.

The OR of women, compared to men, for visits to medical practitioners, intake of ≥3 medications and overall utilization of health-care services fell to values close to 1 on adjusting for variables of health-care need (Table 3). Moreover, the association of home visits with gender lost statistical significance after adjustment for such variables (Table 3). Controlling solely for variables of lifestyle, functional status or cognitive deficit failed to modify materially the OR for any variable of utilization of health services. The variables of need that contributed most to gender differences in the use of services were the number of chronic diseases and HRQL. On adjusting for the number of chronic diseases, the OR (95% CL) of women versus men for ingestion of ≥3 medications was 1.24 (1.06–1.45), and the OR for overall utilization of health-care services was 1.16 (0.95–1.41). After adjustment for HRQL, the OR was 1.03 (0.89–1.21) for visits to medical practitioners, 1.24 (0.98–1.58) for home medical visits, 0.71 (0.58–0.87) for hospitalization, 1.14 (0.97–1.33) for intake of ≥3 medications, and 1.09 (0.89–1.33) for overall utilization of the above services.

When simultaneous adjustment was made for number of chronic diseases and HRQL, the reduction in crude OR was similar and, in the case of hospitalization, even greater than that observed after simultaneous adjustment for all need variables (Table 3). This was because need variables included tobacco, whose use is substantially higher in men than in women, and because the tobacco-adjusted OR for hospitalization of women versus men was 1.19 (95% CL: 0.92–1.54). It should be stressed that, after adjustment for number of chronic diseases and HRQL, the OR for hospitalization of women versus men was 0.68 (95% CL: 0.56–0.84), suggesting that, given equal need, women make less use of hospital services (Table 3).

Adjustment for all factors enabling health-service use led to a reduction in OR for visits to medical practitioners, home visits, intake of ≥3 medications and overall utilization of health-care services (Table 3). In fact, OR for visits to medical practitioners and overall utilization of health-care services took values close to 1 after adjustment for enabling factors. However, OR for home visits and medication intake still showed a statistically significant excess use by women versus men after adjustment. Lastly, OR for influenza vaccination was reduced to 0.77 (95% CL: 0.64–1.02) on adjusting for enabling factors. However, the value of the adjusted OR was included in the 95% confidence interval of the crude OR. Thus, this result should not be overemphasized.

## Discussion

Spanish women aged 60 years and over visit to medical practitioners, receive home medical visits, and use a high number of medications more frequently than men. This is in part explained by variables associated with the need for health services and by factors enabling their use. When the variables that form these groups of factors are considered individually, the number of chronic diseases and HRQL are the variables that contribute mostly to gender differences in the use of such services. Furthermore, after adjusting for these two need factors, a certain inequality was observed in hospital admission in that it proved less frequent among women.

Results on higher female morbidity and its link with greater utilization of health-care services vary across studies. Portraid *et al *reported that age and chronic morbidity were the most important determinants of long-term care need. In addition, after controlling for age and number of chronic diseases, women were institutionalized less frequently than men [33]. Ladwig *et al *described a positive relationship between self-reporting of 24 symptoms and overall utilization of health services, including frequency of medical visits, hospitalization in the preceding 12 months, and number and frequency of medications taken. However, given the same number of reported symptoms, women used health-care services more frequently than did men, except in the category of more than 6 symptoms [6]. In a study on individuals aged 70 years and over, which adjusted for 8 chronic diseases and degree of functional limitation in basic and instrumental activities of daily living, no gender differences were observed in the number of medical contacts in the preceding 2 years, though women required home health-care more frequently than did men. Nevertheless, men were admitted to hospital and used ambulatory surgery services more frequently than women [15]. In our study, adjustment for number of chronic diseases reduced substantially the gender differences in medications taken and overall utilization of health services, thereby partly confirming results of earlier studies.

Our results show that HRQL could account in part for differences between men and women in the frequency of visits to medical practitioners, home medical visits, hospital admission, the number of medications taken, and overall utilization of health-care services. The worse self-perceived health of females, as compared with males, would partly justify their greater use of a number of health-care services, such as visits to general practitioners and use of diagnostic procedures [9]. Although self-reported health is an important predictor of utilization of some health services [11,14,34], only a few studies have specifically assessed the influence of HRQL on gender differences in services use [9].

It is noteworthy that neither functional status nor cognitive deficit explains an appreciable part of gender differences in the utilization of health-care services. Nevertheless, it is plausible that their contribution may be greater to differences in the use of social services or long-term care. In other studies, functional limitation was related to contact with general practitioners and hospitalization in the preceding year [8,13,18], and to women receiving more home medical visits and men being hospitalized or undergoing ambulatory surgery more frequently.[15] On the other hand, no association has been found between degree of cognitive deterioration and health-service use by individuals aged 65 years and over, though such an association has been found with utilization of specific social services [35].

Lifestyles have been linked to state of health and health-service use [21]. Although a positive relationship has been established between BMI and utilization of various health-care services, such as visits to medical practitioners, number of medications prescribed [36], and, particularly in the case of women, visits to hospital and other health professionals [37,38], we have not found studies examining the contribution of BMI or physical activity to gender differences in health-service use. Smoking and alcohol consumption also influence frequency of utilization of health services [21,39,40]. However, the contribution of these behaviors to different patterns of health-service use by women and men has, to our knowledge, never been studied systematically. Our results do not show that the studied lifestyles exert a substantial influence on gender differences in the utilization of health-care services among the elderly.

The contribution of enabling factors to gender differences in health-service use has been addressed in a number of studies [11,15,41]. The respective social roles adopted by men and women have a relevant influence on the greater tendency among women to contact a medical practitioner about a chronic disease. After adjustment for the level of financial and familial responsibility and for employment status, gender differences decreased in the proportion of chronic health problems which entail visits to the medical practitioner [42]. Bertakis *et al *observed that gender differences in the use of different health services (visits to general practitioners, visits to medical specialists, visits to emergency services and use of diagnostic services) were reduced after controlling for educational level, income and race [9]. These findings are consistent with our results for all enabling factors as a whole. In addition, it seems that social network may modify the frequency of use of some health services [18]. In our study, however, the variables that assessed subjects' social network had no independent influence on the unequal utilization of health-care services by elderly men and women.

Certain methodological characteristics of our study must be borne in mind. First, the cross-sectional design does not allow for causal interpretation of the results. Second, the study data are self-reported. Nevertheless, women have not been found to report trivial conditions and mental disorders more frequently than men [43]. Furthermore, there is evidence of the reliability of self reporting of lifestyles [44], chronic diseases [45] and utilization of health-care services [46-48]. Although there is a certain degree of underestimation of health-service use, both after very long recall periods and among hyper-users, there is no evidence of recall errors being different in men and women, so that our results are probably conservative. Third, though we studied a good number of health services, they are not a comprehensive indicator of health-service use. Other studies include other health services, such as telephone consultation [8,34], diagnostic tests [8,9], dental care [14,18] and ambulatory surgery [15]. Moreover, we do not know the reasons for visits to medical practitioners and home medical visits which, because of their diversity (issue of prescriptions, therapeutic or preventive services, scheduled or emergency attention, etc.), might well influence the results obtained. Lastly, no account has been taken of some variables related to the attitude adopted to health problems, the way of self-reporting health status to physicians, and the social structure and the role of women and men, which may in part explain gender differences in the utilization of health-care services [11,18]. Among these are the degree of concern and interest in health, the type of individual response to a specific symptom, paid and non-paid employment, length of the work day (full versus half-day), family demands or burdens, possession of material resources, availability of private medical insurance, and income level.

## Conclusion

Spanish women aged 60 years and over visit to medical practitioners, receive home medical visits, and use a high number of medications more frequently than men. This is in part explained by variables associated with the need for health services and by factors enabling their use. The factors that best explain the greater utilization of health-care services by elderly women versus men are the number of chronic diseases and HRQL. After adjustment for these two need factors, a certain inequality was observed in hospital admission in that it proved less frequent among women. These findings have important implications for health care organizations that seek to provide equal care for both men and women.

## Competing interests

The author(s) declare that they have no competing interests.

## Authors' contributions

FRA, PGC and ARS contributed to the conception and design of the study and to statistical analysis. ARS and FRA wrote the first draft of the manuscript. All authors participated in the interpretation of data for important intellectual content, and revised and approved the final version of the manuscript.

## Pre-publication history

The pre-publication history for this paper can be accessed here:



## Figures and Tables

**Table 1:** Predisposing, need and enabling factors of health-care services use by gender.

	**% women **(n = 1672)	**% men **(n = 1358)	**p**
**Predisposing Factors**			
Age (years)			
- 60–64	26.27	25.33	<0.0001
- 65–69	17.22	24.16	
- 70–74	21.35	22.74	
- 75–79	14.91	14.99	
- 80 and over	20.26	12.79	
Head of family	30.20	93.67	<0.0001
			
**Need Factors**			
Physical activity			
- Sedentary	49.67	34.52	<0.0001
- Occasional	48.03	61.42	
- Regular	2.30	4.06	
Body mass index (kg/m^2^) (%)			
- < 25	22.96	18.86	<0.0001
- 25–29.9	37.48	50.36	
- 30	39.56	30.77	
Tobacco use			
- Never	93.99	30.52	<0.0001
- Ex-smoker	3.87	49.13	
- Smoker	2.14	20.35	
Alcohol consumption			
- Abstainer	70.49	24.99	<0.0001
- Ex-drinker	6.13	18.15	
- Moderate drinker	20.82	38.09	
- Heavy drinker	2.56	18.77	
Arterial hypertension	69.28	66.93	0.173
Number of chronic diseases			
- 0	17.58	31.13	<0.0001
- 1	38.89	38.56	
- 2	27.00	19.67	
- 3 or more	16.53	10.63	
Independent in IADL	59.30	72.90	<0.0001
Cognitive deficit (Cognition Mini-exam)	25.30	14.94	<0.0001
Health-related quality of life (SF-36)			
- Summary Physical Index (mean and SD)	42.66 (10.43)	46.19 (10.75)	<0.0001
- Summary Mental Index (mean and SD)	47.43 (11.19)	52.40 (9.81)	<0.0001
			
**Enabling Factors**			
Educational level			
- No formal education	55.56	44.29	<0.0001
- Primary	35.29	36.15	
- Secondary	6.47	13.29	
- University	2.67	6.27	
Marital status: married	47.71	81.56	<0.0001
Head-of-family employment status			
- Employed	13.37	33.15	<0.0001
- Unemployed	0.82	3.53	
- Retired/Housewife	85.81	63.32	
Social network			
- Lives alone	23.13	8.64	<0.0001
- Sees family daily or almost daily	54.16	51.93	0.220
- Sees friends or neighbours daily or almost daily	81.39	88.24	<0.0001

IADL: instrumental activities of daily living. SD: standard deviation

**Table 2:** Utilization of health-care services by gender.

**Health service**	**% women**	**% men**	**p**
**Visit to medical practitioner ≥once per month**	41.97	36.85	0.004
**Home visit ≥once per year**	15.07	9.58	<0.0001
**Hospital admission in the preceding year**	17.34	18.12	0.573
**Influenza vaccination in the most recent campaign**	54.75	56.14	0.443
**Current intake of ≥3medications**	52.19	41.40	<0.0001
**Overall utilisation (≥1 of the above health-care services)**	84.83	79.49	<0.0001

**Table 3:** Odds ratios (95% confidence limits) of health-care services use by women versus men.

**Health Service**	**Crude OR**	**OR adjusted for predisposing factors**	**OR adjusted for need factors**	**OR adjusted for chronic diseases and HRQL**	**OR adjusted for enabling factors**
**Visit to medical practitioner ≥once per month**	1.24 (1.07–1.44)**	1.09 (0.90–1.33)	0.90 (0.72–1.12)	0.99 (0.84–1.15)	0.97 (0.80–1.19)
**Home visit ≥once per year**	1.67 (1.34–2.10)**	1.31 (0.98–1.75)	1.26 (0.88–1.81)	1.24 (0.97–1.58)	1.31 (1.00–1.75)*
**Hospital admission in the preceding year**	0.95 (0.79–1.14)	0.90 (0.71–1.16)	0.85 (0.64–1.14)	0.68 (0.56–0.84)**	0.79 (0.61–1.02)
**Influenza vaccination in the most recent campaign**	0.94 (0.82–1.09)	0.91 (0.74–1.10)	0.76 (0.61–0.94)*	0.85 (0.73–0.99)*	0.77 (0.64–0.94)*
**Current intake of ≥3 medications**	1.54 (1.34–1.79)**	1.35 (1.11–1.63)**	1.05 (0.84–1.33)	1.04 (0.88–1.23)	1.24 (1.02–1.51)*
**Overall utilisation (≥1 of the above health-care services)**	1.44 (1.20–1.74)**	1.19 (0.92–1.54)	0.98 (0.74–1.30)	1.00 (0.82–1.23)	0.92 (0.71–1.20)

**OR**: Odds ratio. **HRQL**: Health-related quality of life. **Predisposing factors**: Age, and head-of-family status. **Need factors**: Physical activity, tobacco, alcohol consumption, arterial hypertension, number of chronic diseases, Lawton and Brody index of limitations in independent activities of daily living, cognitive deficit with the Cognition Mini-Exam, and physical and mental summary scores of the SF-36. **Enabling factors**: Educational level, marital status, head-of-family employment status, and social network. * p < 0.05; **p < 0.01.
